# Reduced Effectiveness and Comparable Safety in Biweekly *vs.* Weekly PEGylated Recombinant Human Growth Hormone for Children With Growth Hormone Deficiency: A Phase IV Non-Inferiority Threshold Targeted Trial

**DOI:** 10.3389/fendo.2021.779365

**Published:** 2021-11-25

**Authors:** Chengjun Sun, Biao Lu, Yu Liu, Yaqin Zhang, Haiyan Wei, Xu Hu, Pei Hu, Qian Zhao, Yanling Liu, Kan Ye, Kan Wang, Zaiyan Gu, Zheng Liu, Jin Ye, Hongxiao Zhang, Hong Zhu, Zhihong Jiang, Yanjie Liu, Naijun Wan, Chengming Yan, Jianying Yin, Lirong Ying, Feng Huang, Qingjin Yin, Li Xi, Feihong Luo, Ruoqian Cheng

**Affiliations:** ^1^ Department of Pediatric Endocrinology and Inherited Metabolic Diseases, Children’s Hospital of Fudan University, Shanghai, China; ^2^ Department of Pediatrics, General Hospital of Ningxia Medical University, Yinchuan, China; ^3^ Department of Endocrine and Genetic Metabolism, Maternal and Child Health-Care Hospital in Guiyang, Guiyang, China; ^4^ Department of Child Health, Maternal and Child Health Care Hospital of Hainan Province, Haikou, China; ^5^ Department of Pediatric Endocrinology and Inherited Metabolic Diseases, Henan Provincial Hospital, Affiliated to Zhengzhou University, Zhengzhou, China; ^6^ Department of Pediatrics, Lu’an People’s Hospital, Lu’an, China; ^7^ Clinical Pharmacology Research Center, Peking Union Medical College Hospital, State Key Laboratory of Complex Severe and Rare Diseases, National Medical Products Administration (NMPA) Key Laboratory for Clinical Research and Evaluation of Drug, Beijing Key Laboratory of Clinical Pharmacokinetics and Pharmacodynamics (PK & PD) Investigation for Innovative Drugs, Chinese Academy of Medical Sciences & Peking Union Medical College, Beijing, China; ^8^ Department of Pediatrics, The Second Affiliated Hospital of Nanchang University, Nanchang, China; ^9^ Department of Child Health, The Affiliated Suzhou Hospital of Nanjing Medical University, Suzhou, China; ^10^ Department of Pediatrics, Jinhua Hospital, Zhejiang University School of Medicine, Jinhua, China; ^11^ Department of Pediatrics, Jiaxing First Hospital, Jiaxing, China; ^12^ Department of Pediatrics, Tai’an Maternal and Child Health Care Hospital, Tai’an, China; ^13^ Department of Pediatrics, Jiangsu Province Hospital of Chinese Medicine, Nanjing, China; ^14^ Department of Pediatrics, Second Hospital of Lanzhou University, Lanzhou, China; ^15^ Department of Pediatrics, The First People’s Hospital of Changzhou, Changzhou, China; ^16^ Department of Pediatrics, The First Affiliated Hospital of Henan University of Science and Technology, Luoyang, China; ^17^ Department of Pediatrics, Inner Mongolia People’s Hospital, Hohhot, China; ^18^ Department of Pediatrics, Jishuitan Hospital, Beijing, China; ^19^ Department of Pediatrics, Anhui Province Maternity and Child Health Hospital, Anhui Medical University Maternal and Child Health Clinic College, Hefei, China; ^20^ Department of Pediatrics, Hebei General Hospital, Shijiazhuang, China; ^21^ Department of Pediatrics, Cixi People’s Hospital, Cixi, China; ^22^ Department of Pediatrics, Affiliated Hospital of Nantong University, Nantong, China; ^23^ Department of Internal Medicine, Chengdu Children’s Specialized Hospital, Chengdu, China

**Keywords:** growth hormone deficiency, PEGylated recombinant human growth hormone, PEG-rhGH, IGF-2, children

## Abstract

**Context:**

Long-acting recombinant human growth hormone (rhGH) has transformed growth hormone deficiency (GHD) treatment. However, the possibility and rationality for flexible time regimen are pending.

**Objective:**

We studied the efficacy of biweekly *versus* weekly PEGylated rhGH (PEG-rhGH) therapy in GHD children.

**Design, Setting, and Patients:**

This multicenter, phase IV trial with a non-inferiority threshold ≥20% enrolled 585 Tanner stage I GHD children.

**Intervention:**

Subjects randomly received 0.20 mg/kg once-weekly or biweekly PEG-rhGH, or 0.25 mg/kg.w rhGH once daily for 26 weeks.

**Main Outcome Measure:**

The primary outcome was height SD scores for chronological age (HtSDS_CA_) at week 26 and safety measurements including adverse events (AEs), IGF-2, and IGFBP-2 changes.

**Results:**

At week 26, the median HtSDS_CA_ changed from −2.75, −2.82, and −2.78 to −2.31, −2.43, and −2.28 with weekly and biweekly PEG-rhGH, and daily rhGH, respectively. The difference in HtSDS_CA_ was 0.17 ± 0.28 between weekly and biweekly PEG-rhGH, and 0.17 ± 0.27 between daily rhGH and biweekly PEG-rhGH, failing the non-inferiority threshold. Nevertheless, the height velocity of children receiving biweekly PEG-rhGH reached 76.42%–90.34% and 76.08%–90.60% that of children receiving weekly PEG-rhGH and daily rhGH, respectively. The rate of AEs was comparable among the groups. No statistical difference was observed in IGF-2 and IGFBP-2 levels among the groups. IGFBP-2 levels decreased over time in all groups, with no notable difference in IGF-2 and IGFBP-2 changes among the three treatment groups.

**Conclusions:**

Although notably promoted height velocity, biweekly PEG-rhGH failed the non-inferiority threshold as compared with either weekly PEG-rhGH or daily rhGH. Compared with short-term rhGH, long-acting PEG-rhGH did not significantly increase tumor-associated IGF-2 and IGFBP-2 expressions.

**Clinical Trial Registration:**

clinicaltrials.gov, identifier NCT02976675.

## 1 Introduction

Growth hormone deficiency (GHD) is a rare cause of childhood short stature and characterized by a diminished height velocity (HV) and/or third percentile below the mean height of a normal population with the same chronological age and sex. A survey of about 100,000 Chinese children aged between 6 and 15 years revealed an incidence of 1/8,646 for GHD (1). The main treatment for GHD is recombinant human growth hormone (rhGH), with the primary goal in GHD children to normalize height before adulthood.

Due to its short half-life (approximately 0.5 to 2 h after a subcutaneous injection), rhGH is required to be administered as a daily injection (2). Frequent injections cause distress to the children, therefore reducing compliance. Long-acting rhGH formulations with a prolonged half-life and reduced injection frequencies could enhance children’s compliance with rhGH treatment. PEGylated rhGH (PEG-rhGH) is one of the long-acting rhGH formulations that have a prolonged half-life of elimination. PEG-rhGH (Jintrolong^®^) has been recently approved for the treatment of children with GHD by China National Medical Products Administration (NMPA). Previous pharmacokinetics (PK) study showed that PEG-rhGH had a half-life of 32 ± 5 h and still maintained a high concentration (7.9 ng/ml) in circulation after five half-lives, supporting a weekly dosing schedule (3). A phase III trial in 343 children with GHD demonstrated the safety and efficacy of weekly Jintrolong^®^ (0.2 mg/kg/week) for 25 weeks that is non-inferior to daily rhGH (0.25 mg/kg/week).

The promotion of linear growth by GH is primarily mediated by insulin-like growth factor-1 (IGF-1) (4). Although another phase I trial of PEG-rhGH showed that IGF-1 levels declined faster in children with GHD than in healthy adults after PEG-rhGH injection (IGF-1 decline to approximately 80% of peak IGF-1 at 5 days after PEG-rhGH administration), the trial showed that the area under the curve (AUC) of IGF-1 of PEG-rhGH (0.2 mg/kg/week) was 1.1- to 1.3-fold that of an equivalent dose of rhGH (4–6). In addition, phase II/III trials revealed that the increase in height SD scores (HtSDS) with weekly PEG-rhGH for 25 weeks was 84%–100% that of rhGH (6). These data suggest that PEG-rhGH can be administered with longer intervals while still maintaining comparable efficacy. Currently, it remains unclear whether longer dosing interval such as PEG-rhGH every other week offers comparative efficacy and safety *versus* once-weekly PEG-rhGH, especially among children with GHD. Our unpublished PK data showed that the serum IGF-1 levels were significantly elevated after multiple subcutaneous injections of PEG-rhGH (0.2 mg/kg/week) every other week (138.0 ± 67.6 *vs.* 102.0 ± 46.2 ng/ml before PEG-rhGH).

To optimize PEG-rhGH dosing schedule and provide rationales for further exploration of feasibility of PEG-rhGH injection once every other week, we undertook this multicenter, phase IV, randomized, open-label, parallel-group, non-inferiority trial to compare the efficacy and safety of PEG-rhGH once every other week *versus* PEG-rhGH once-weekly and once-daily rhGH for Chinese children with GHD.

## 2 Materials and Methods

### 2.1 The Study Population

The phase IV, randomized, open-label, parallel-group, non-inferiority trial was conducted at 31 medical centers across China (**Appendix I**) between January 2015 and December 2017. The study enrolled Tanner stage I prepubertal children with confirmed GHD who were aged at least 3 years. The inclusion criteria were 1) height below the third percentile in reference to children of the same chronological age and sex; 2) HV ≤5.0 cm/year; 3) serum GH peak levels <10.0 ng/ml by two different stimulation tests; 4) bone age ≤9 years (girls) or ≤10 years (boys), and bone age delayed for at least 1 year compared with the patient’s chronological age; and 5) not treated by rhGH in the preceding 6 months. Brain and pituitary MRI was performed in all patients to assist the etiological diagnosis. Patients with multiple pituitary hormone deficiencies were included after stable hormone replacement therapy (except GH) for at least 3 months. The exclusion criteria were as follows: 1) renal or hepatic impairment (alanine aminotransferase >2 times the upper normal limit or creatine greater than the upper normal limit); 2) serum positive anti-HBc antibody, HBsAg, or HBeAg; 3) hypersensitivity or allergy to PEG-rhGH; 4) severe cardiac, pulmonary, hematological diseases, or systemic infections, immunocompromisation, diabetes, and familial history of malignancies; 5) other known diseases presenting growth abnormalities (Turner syndrome, delayed constitutive pubertal development, or Laron syndrome, etc.); 6) congenital skeletal development abnormalities or scoliosis; and 7) active clinical trial participants in the preceding 3 months.

The study protocol adhered to the SPIRIT statement and was approved by the ethics committee of each participating center (7). The trial is registered with clinicaltrials.gov (NCT02976675) and was conducted in accordance with the International Conference on Harmonisation Guidelines for Good Clinical Practice and the Declaration of Helsinki. The reporting of the study adhered to the CONSORT statement (8). Informed consent was obtained in writing from age-appropriate children and their parents or legal guardians.

### 2.2 Randomization and Treatment

Eligible children were randomized in a 1:1:1 ratio using computer-generated randomization sequence in blocks to receive subcutaneous injections of PEG-rhGH (Jintrolong^®^, GeneScience Pharmaceuticals, Changchun, China) at a dose of 0.20 mg/kg once weekly (QW) or once every other week (QOW), or 0.25 mg/kg rhGH (Jintropin AQ^®^, GeneScience Pharmaceuticals) once daily (QD) for 26 weeks. During the trial, patients who received concomitant administration of GnRH analogues, androgenic hormones, anabolic androgenic steroids, or other medications that may have an effect on growth were excluded.

### 2.3 Patient Evaluation

All patients were assessed at baseline and at 4, 13, and 26 weeks after treatment initiation. At each assessment, height and weight were measured, and blood samples were tested for serum IGF-1 and IGF binding protein-3 (IGFBP-3), cortisol, free thyroxine (T4), thyroid-stimulating hormone (TSH), calcium, phosphate, lipids, glycated hemoglobin (HbA1c), fasting insulin, fasting plasma glucose (FPG), and homeostasis model assessment of insulin resistance (HOMA-IR), complete blood count, and liver and renal function. In addition, we measured serum levels of IGF-2 and IGFBP-2 (Quantikine Immunoassay, R&D Systems, USA) using leftover specimens at baseline and weeks 4, 12, and 26 at a centralized laboratory. Bone age radiographs were obtained at baseline and week 26 and read centrally at the Children’s Hospital of Fudan University by two experienced, blinded radiologists using the TW3 method. The compliance of the patients was determined by patient diaries and vial counting. During the trial, adverse events (AEs) were graded and summarized at baseline and weeks 4, 12, and 26 according to the National Cancer Institute (NCI) Common Terminology Criteria for Adverse Events (CTCAE) version 4.03 and coded using MedDRA 22.0.

### 2.4 Statistical Analysis

Based on the phase III trial, assuming less than 20% difference (δ ≤ 0.2) in changes in HtSDS for chronological age (HtSDS_CA_) at week 26 from baseline between PEG-rhGH 0.20 mg/kg/2w and PEG-rhGH 0.20 mg/kg/week and α = 0.015, a sample size of 112 children for each group was required to guarantee a power of 90% (6). In the phase III trial, rhGH 0.25 mg/kg/week had a slightly lower efficacy than PEG-rhGH 0.20 mg/kg/week, so this sample size would guarantee less than 20% difference in efficacy between PEG-rhGH 0.20 mg/kg/2w and rhGH 0.25 mg/kg/week. Assuming a dropout rate of 20%, a sample size of at least 150 children was required for each group. The study planned to enroll a target population of 600 children, with 200 children in each group.

Statistical analyses were prespecified, followed the intention-to-treat (ITT) principle, and undertaken using the SAS software package, version 9.4 (SAS Institute Inc., Cary, NC). Quantitative variables were expressed in mean and SD if normally distributed, paired *t*-test or Wilcoxon rank-sum test was used for comparing differences between the baseline and treatment, and *t*-test or ANOVA was used for comparison between groups. Non-normally distributed data were expressed as median and interquartile range (IQR), and non-parametric tests were used for comparison between groups. Categorical variables were described in frequency and percentage and analyzed by chi-square (χ^2^) test including Cochran–Mantel–Haenszel (CMH)-χ^2^ test or Fisher’s exact test.

The primary efficacy end point was HtSDS_CA_ at week 26. HtSDS_CA_ change from baseline at week 26 was compared and analyzed using the analysis of covariance (ANCOVA) model in which the baseline variable was used as a covariate, taking into consideration the center effect. Last observation carried forward (LOCF) was used for the study end points for children who did not receive full evaluation for efficacy. Non-inferiority was examined using two sample *t*-test. The non-inferiority threshold (Δ) was set at no lower than 20%, and non-inferiority was established if the upper limit (UL) of 97.5%CI (one-sided) was smaller than the non-inferiority threshold. One-sided *p* ≤ 0.025 was considered statistically significant. The secondary efficacy end points included annualized HV, IGF-1 SDS, IGF-1/IGFBP-3 molar ratio, and bone maturation. Changes in the secondary efficacy end points from baseline were compared using ANCOVA. *p* ≤ 0.05 (two-sided) was considered as statistically significantly different.

Safety data were analyzed mainly using descriptive statistics. χ^2^ test including CMH-χ^2^ test was used to compare the incidence of AEs between groups.

## 3 Results

### 3.1 Patient Demographic and Baseline Characteristics

**Figure 1:**
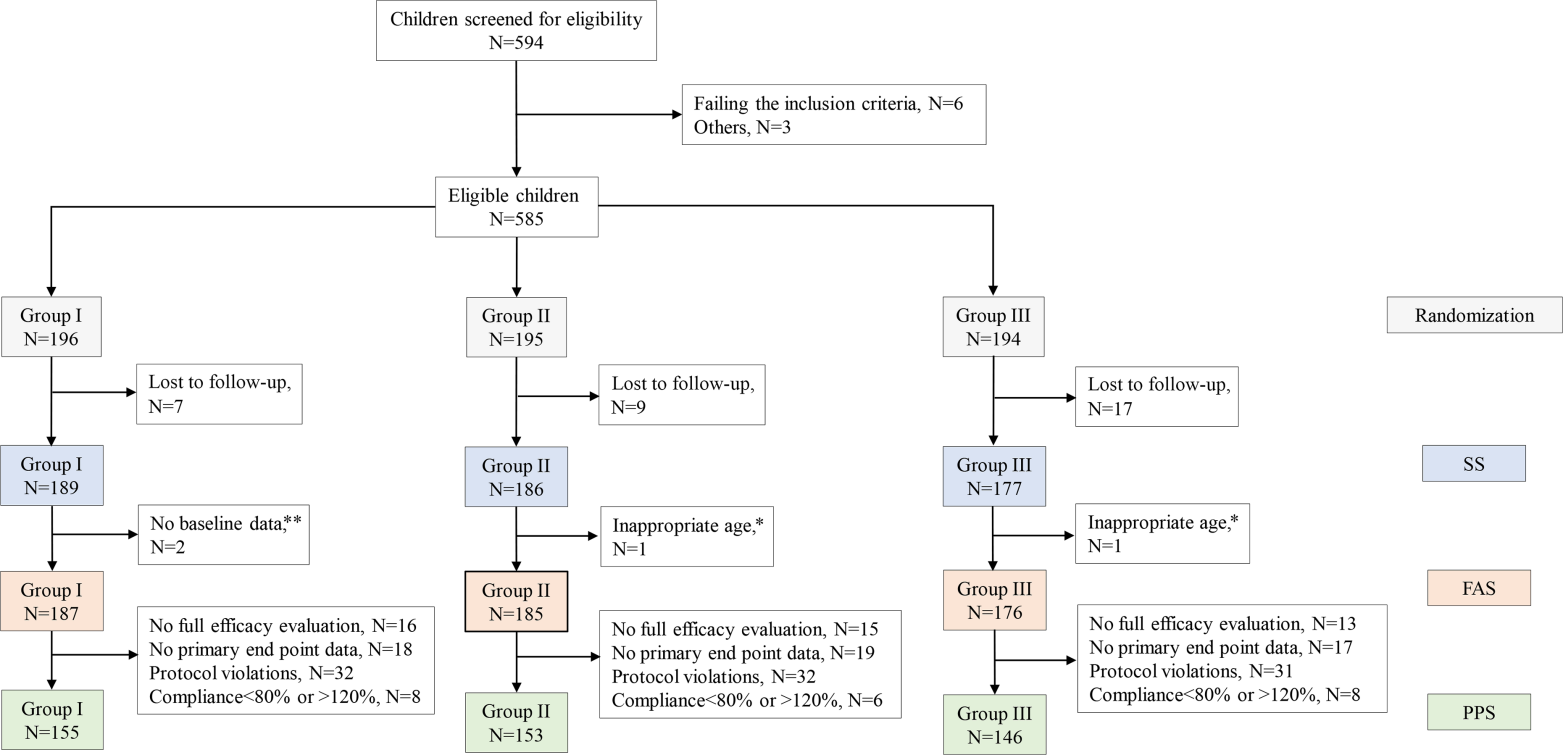
The study flowchart. Note: A subject may be excluded from the per-protocol set (PPS) due to more than one cause. *Participant’s age > 18 years. **Incomplete height record at baseline.

The study flowchart is shown in **Figure 1**. The study screened 594 children for eligibility; we excluded six children who did not meet the inclusion criteria and three children due to other causes. A total of 585 children underwent randomization, with 196, 195, and 194 children in groups QW, QOW, and QD, respectively. We further excluded 33 children due to lost to follow-up, two children without baseline data, and two children who were not of the appropriate age. Finally, 548 children were included. The three groups were comparable in the demographic and baseline variables (**Table 1**).

**Table 1:** Demographic and baseline of the study population-FAS.

Intervention group				
Variables	QW	QOW	QD	*p*
N	187	185	176	
Mean age ± SD, years	7.8 ± 2.8	7.6 ± 2.7	8.0 ± 2.9	0.530
Male gender, n (%)	135 (72.2%)	124 (67.0%)	122 (69.5%)	0.556*
Mean bone age ± SD, years	5.6 ± 2.7	.5.4 ± 2.6	5.7 ± 2.6	0.618
Mean height ± SD, cm	113.5 ± 14.2	111.7 ± 14.0	112.9 ± 12.9	0.427
Mean body weight ± SD, kg	20.7 ± 7.1	19.9 ± 6.0	20.4 ± 6.1	0.562
Mean BMI ± SD, kg/m^2^	15.6 ± 2.1	15.7 ± 2.3	15.8 ± 1.9	0.482
Median Ht SDS_CA_ (Q1, Q3)	−2.75 (−3.10, −2.40)	−2.82 (−3.33, −2.39)	−2.78 (−3.37, −2.42)	0.284^#^
Median IGF-1 (Q1, Q3), ng/ml	100.0 (58.7, 155.0)	94.5 (59.4, 142.0)	88.40 (62.0, 151.0)	0.785
Median IGF-1 SDS (Q1, Q3)	−1.13 (−1.69, −0.38)	−1.04 (−1.62, −0.51)	−1.15 (−1.58, −0.72)	0.633^#^
Median IGFBP-3 (Q1, Q3), mg/ml	3.29 (2.58, 4.38)	3.46 (2.36, 4.10)	3.35 (2.72, 4.32)	0.945
Mean FPG ± SD, mmol/L	4.6 ± 0.6	4.6 ± 0.6	4.7 ± 0.5	0.251
Mean HbA1c ± SD, %	5.2 ± 0.4	5.1 ± 0.4	5.2 ± 0.4	0.730
Median TSH (Q1, Q3), mU/L	2.70 (1.90, 3.84)	2.62 (1.90, 3.81)	2.68 (1.94, 4.25)	
Mean IGF-2 ± SD, ng/ml	326.0 ± 109.5	322.7 ± 130.8	345.5 ± 154.9	0.320^
Median IGFBP-2 (Q1, Q3), ng/ml	254.2 (190.4, 334.9)	259.7 (187.8, 338.7)	224.2 (171.3, 310.3)	0.082^#^

BMI, body mass index; FPG, fasting plasma glucose; GH, growth hormone; HbA1c, hemoglobin A1c; Ht, height; IGF, insulin-like growth factor; IGFBP-3, insulin growth factor binding protein-3; SDS, SD scores; TSH, thyroid-stimulating hormone.

*χ^2^ test.

^#^Kruskal–Wallis test.

^ANOVA.

### 3.2 Height SD Scores for Chronological Age

**Figure 2:**
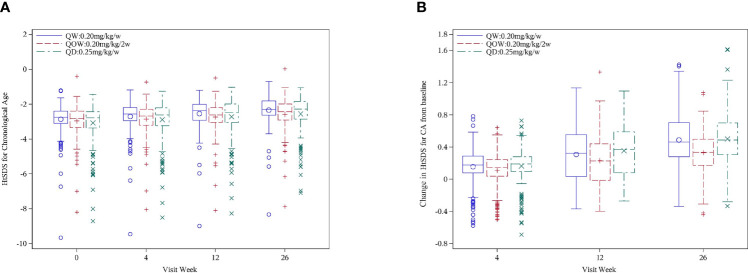
**(A)** Height SD scores for chronological age (HtSDSCA) for PEGylated recombinant human growth hormone (PEG-rhGH) every other week (QOW), weekly PEG-rhGH (QW), and daily rhGH (QD) at baseline and weeks 4, 12, and 26. **(B)** HtSDS_CA_ change at weeks 4, 12, and 26 from baseline for PEG-rhGH QOW, PEG-rhGH QW, and rhGH QD.

HtSDS_CA_ was comparable among the three groups at baseline and week 4 (**Table 1**). A statistically significant difference was also observed at week 26 in median HtSDS_CA_ among the three groups: −2.31 (Q1, Q3, −2.63, −1.81), −2.43 (Q1, Q3 −2.91, −1.98), and −2.28 (Q, Q3, −2.85, −1.83) for groups QW, QOW, and QD, respectively (**Figure 2A**). Meanwhile, there was a statistically significant difference in HtSDS_CA_ change at weeks 4, 12, and 26 from baseline among the three groups (**Figure 2B**). At week 26, the median increase in HtSDS_CA_ in groups QW, QOW, and QD were 0.52 (Q1, Q3, 0.34, 0.74), 0.38 (Q1, Q3, 0.25, 0.52), and 0.51 (Q1, Q3, 0.35, 0.73), respectively.

The difference in HtSDS_CA_ at week 26 was 0.17 ± 0.28 (97.5%CI UL 0.23) between groups QW and QOW, and 0.17 ± 0.27 (97.5%CI UL 0.23) between groups QOW and QD, and the UL of 97.5%CI was 0.23 in the difference in HtSDS_CA_ at week 26 between both groups QW and QOW and between groups QOW and QD, and was larger than the non-inferiority threshold (Δ = 0.11), thus failing the non-inferiority test (**Table 2**). After group QOW was stratified by stimulated peak GH levels (<7 *vs.* ≥7 ng/ml) on provocative tests, non-inferiority was not established either (**Table 3**).

**Table 2:** Non-inferiority of PEG-rhGH every other week (QOW) *versus* weekly PEG-rhGH (QW) and daily rhGH (QD).

Group	HtSDS_CA_			Annualized HV		
	Mean (SD)	97.5%CI	Δ	Mean (SD)	97.5%CI	Δ
**QW**	0.55 (0.32)	0.49, 0.60		10.82 (2.67)	10.35, 11.28	
**QOW**	0.37 (0.24)	0.33, 0.42		8.98 (2.10)	8.62, 9.35	
*DIFF(QW − QOW)*	0.17 (0.28)	−Infinite, 0.23	0.11	1.83 (2.40)	−Infinite, 2.35	2.16
**QD**	0.54 (0.29)	0.49, 0.60		10.82 (2.83)	10.32, 11.33	
**QOW**	0.37 (0.24)	0.33, 0.42		8.98 (2.10)	8.62, 9.35	
*DIFF(QD − QOW)*	0.17 (0.27)	−Infinite, 0.23	0.11	1.84 (2.48)	−Infinite, 2.38	2.16
QW	0.55 (0.32)	0.50, 0.59		10.82 (2.67)	10.41, 11.22	
QD	0.54 (0.29)	0.50, 0.59		10.82 (2.83)	10.38, 11.27	
*DIFF(QW − QD)*	−0.01 (0.34)	−0.08, 0.06	0.00	−0.01 (2.75)	−0.60, 0.59	0.00

DIFF, difference; Δ, non-inferiority threshold; PEG-rhGH, PEGylated recombinant human growth hormone; HtSDS_CA_, height SD scores for chronological age; HV, height velocity.

**Table 3:** Non-inferiority of PEG-rhGH every other week (QOW) *versus* weekly PEG-rhGH (QW) and daily rhGH (QD) stratified by GH peak (<7 or ≥7 μg/L).

	HtSDS_CA_		
Group	Mean (SD)	97.5%CI	Δ
**GH peak <7 μg/L**			
QW	0.54 (0.35)	0.46, 0.62	
QOW	0.39 (0.25)	0.33, 0.45	
*DIFF(QW − QOW)*	0.15 (0.31)	−Infinite, 0.24	0.11
QD	0.55 (0.32)	0.48, 0.63	
QOW	0.39 (0.25)	0.33, 0.45	
*DIFF(QD − QOW)*	0.17 (0.29)	−Infinite, 0.26	0.11
QW	0.54 (0.35)	0.47, 0.61	
QD	0.55 (0.32)	0.49, 0.62	
*DIFF(QW − QD)*	−0.01 (0.34)	−0.11, 0.09	0.00
**GH peak ≥7 μg/L**			
QW	0.55 (0.28)	0.48, 0.62	
QOW	0.36 (0.23)	0.29, 0.42	
*DIFF(QW − QOW)*	0.20 (0.26)	−Infinite, 0.28	0.11
QD	0.53 (0.26)	0.46, 0.60	
QOW	0.36 (0.23)	0.29, 0.42	
* DIFF(QD − QOW)*	0.18 (0.24)	−Infinite, 0.26	0.956
QW	0.55 (0.28)	0.49, 0.61	
QD	0.53 (0.26)	0.47, 0.59	
* DIFF(QW − QD)*	0.02 (0.27)	−0.07, 0.11	0.00

DIFF, difference; Δ, non-inferiority threshold; PEG-rhGH, PEGylated recombinant human growth hormone; HtSDS_CA_, height SD scores for chronological age.

In addition, ANCOVA was performed using baseline HtSDS_CA_ and center effects as covariates to compare the least square mean (LSM) of HtSDS_CA_ change at week 26 from baseline among the three groups. The LSM was 0.56, 0.38, and 0.54 for groups QW, QOW, and QD, respectively, with a significant difference in HtSDS_CA_ change by groups (*F* = 21.23, *p* < 0.001) and baseline HtSDS_CA_ (*F* = 33.30, *p* < 0.001) (**Supplementary Table 1**). No significant center effect was observed (*F* = 1.521, *p* = 0.066). Furthermore, Bonferroni correction showed statistical difference in HtSDS_CA_ change between groups QW and QOW, and groups QOW and QD (*p* < 0.05). Stepwise multivariate logistic regression analysis using HtSDS_CA_ change as a dependent variable and group, age, gender, baseline IGF-1, and peak GH levels as independent variables further revealed that group, age, baseline IGF-1, and peak GH levels were significant determinants of HtSDS_CA_ change at week 26 (*p* < 0.05) (**Supplementary Table 2**). Moreover, age, baseline IGF-1, and peak GH levels negatively correlated with HtSDS_CA_ change at week 26.

### 3.3 Secondary Efficacy Measures

#### 3.3.1 Annualized Height Velocity

**Figure 3:**
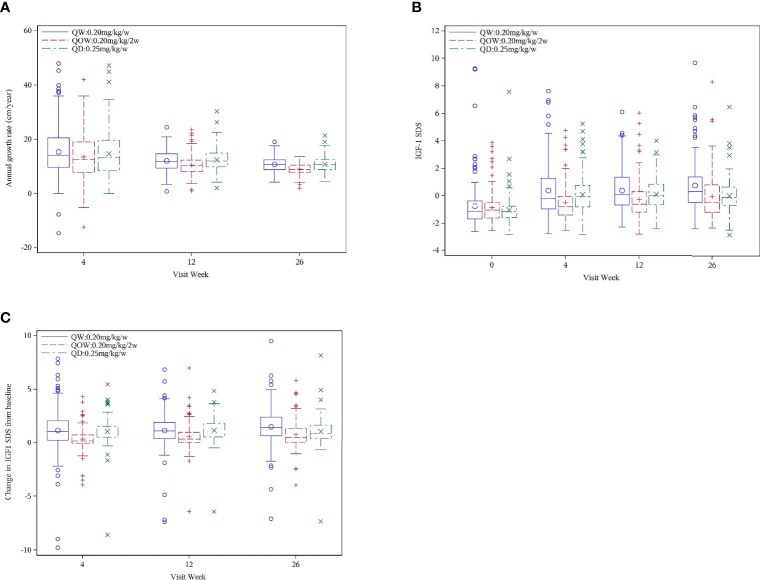
Annualized height velocity (HV) **(A)** and IGF-1 SD scores (SDS) **(B)** for PEGylated recombinant human growth hormone (PEG-rhGH) every other week (QOW), weekly PEG-rhGH (QW), and daily rhGH (QD) at baseline and weeks 4, 12, and 26. IGF-1 SDS change **(C)** at weeks 4, 12, and 26 from baseline for PEG-rhGH QOW, PEG-rhGH QW, and rhGH QD.

Significant difference in annualized HV was observed among the three groups at weeks 12 and 26 (**Figure 3A**). At week 26, groups QW (10.82 ± 2.67 cm/year) and QD (10.82 ± 2.83 cm/year) had higher annualized HV than group QOW (8.98 ± 2.10 cm/year). The difference in annualized HV at week 26 was 1.83 ± 2.40 (97.5%CI UL 2.35) between groups QW and QOW, and 1.84 ± 2.48 (97.5%CI UL 2.38) between groups QD and QOW, and the UL of 97.5%CI in the difference in annualized HV at week 26 between both groups QW and QOW and between groups QOW and QD was greater than the non-inferiority threshold (Δ = 2.16), thus failing the non-inferiority test (**Table 2**). Nevertheless, the HV of group QOW was 76.42%–90.34% that of group QW and 76.08%–90.60% of group QD.

#### 3.3.2 Insulin-Like Growth Factor-1 SD Score

Significant difference in IGF-1 SDS was observed among the three groups at weeks 4, 12, and 26 (**Figure 3B**). At week 26, IGF-1 SDS were 0.29 (Q1, Q3, 0.67, 2.41), −0.47 (Q1, Q3, 0.02, 1.33), and −0.13 (Q1, Q3, 0.41, 1.65) in groups QW, QOW, and QD, respectively. The change in IGF-1 SDS after 26 weeks of treatment was 1.42 (Q1, Q3, 0.67, 2.41), 0.50 (Q1, Q3, 0.02, 1.33), and 0.90 (Q1, Q3, 0.41, 1.65) in groups QW, QOW, and QD, respectively (**Figure 3C**).

### 3.4 Safety

The safety data were available in 552 children. A total of 488 AEs occurred; there was no statistical difference in the incidence of AEs and severe AEs (SAEs) among the three groups (*p* = 0.486; *p* = 0.691, **Table 4**). AEs occurred at least once in 39.7%, 28.0%, and 33.3% of the patients in groups QW, QOW, and QD, respectively (*p* = 0.055). The most common AE was upper respiratory tract infection (27.0%) followed by cough (9.5%) and fever (6.4%) in group QW (**Supplementary Table 3**). In groups QOW and QD, upper respiratory tract infection (30.7% and 31.1%) was the most common AE followed by fever (11.3% and 20.4%) and bronchial infection (5.4% and 2.3%). No AEs led to growth hormone treatment withdrawal in all three groups. In addition, there were no clinically relevant changes from baseline to week 26 in mean HbA1c, FPG, insulin, or HOMA-IR in any of the treatment groups.

**Table 4:** Adverse events (AEs) and severe AEs (SAEs) in the safety set.

	QW (N = 189)	QOW (N = 186)	QD (N = 177)	*p*
**AEs occurring at least once**	75 (39.7%)	52 (28.0%)	59 (33.3%)	0.055
**AEs, n (%)**	170 (90.0%)	169 (90.9%)	149 (84.2%)	0.486
**SAEs**	6 (3.2%)	4 (2.2%)	3 (1.6%)	0.691
**Severity**				0.8591
Mild	122 (54.0%)	117 (48.6%)	104 (49.3%)	
Moderate	17 (7.5%)	17 (7.1%)	15 (7.1%)	
Severe	5 (2.2%)	2 (0.8%)	2 (1.0%)	

QW, once weekly; QOW, once every other week; QD, once daily.

### 3.5 Insulin-Like Growth Factor-2 and Insulin-Like Growth Factor Binding Protein-2

**Figure 4:**
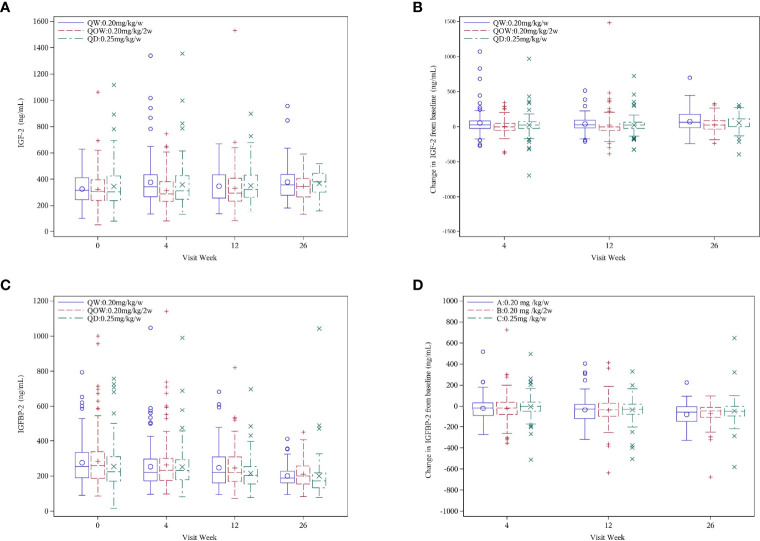
**(A)** IGF-2, **(B)** IGF-2 change, **(C)** IGFBP-2, and **(D)** IGFBP-2 change for the PEGylated recombinant human growth hormone (PEG-rhGH) every other week (QOW), weekly PEG-rhGH (QW), and daily rhGH (QD) groups at baseline and weeks 4, 12, and 26.

The three groups were comparable in the mean baseline IGF-2 and IGFBP-2 levels (**Table 1**). At week 26, no statistical difference was observed in IGF-2 and IGFBP-2 levels among the three groups (**Figures 4A, C**). Except a significant IGF-2 change at week 4 from baseline (Kruskal–Wallis test, *p* = 0.044), there was no notable statistical difference in IGF-2 change at weeks 12 and 26 (Kruskal–Wallis test, *p* = 0.086 and 0.161, respectively) (**Figure 4B**). IGFBP-2 levels decreased over time in all the three groups, but no statistical difference was observed in IGFBP-2 change at weeks 4, 12, and 26 (Kruskal–Wallis test, *p* = 0.153, 0.819, and 0.436, respectively) (**Figure 4D**).

## 4 Discussion

In the current phase IV non-inferiority trial, PEG-rhGH every other week significantly increases HtSDS_CA_ at week 26 and is associated with increased annualized HV and IGF-1 SDS. In addition, PEG-rhGH every other week has a safety profile comparable with that of weekly PEG-rhGH and daily rhGH. Though the study failed to establish non-inferiority of PEG-rhGH every other week to weekly PEG-rhGH or daily rhGH, PEG-rhGH given every other week leads to significant improvement in HtSDS_CA_, annualized HV, and IGF-1 SDS and, therefore, could offer children with GHD a safe alternative to the current once-weekly PEG-rhGH regimen, with a less frequent dosing schedule and a much-reduced medication cost.

GH promotes the linear growth of children primarily *via* the action of stimulating IGF-1, which, as an indicator for bioavailable GH and treatment response, can be used to monitor rhGH treatment. Availability of an effective and safe but less frequent dosing schedule than weekly PEG-rhGH would be desirable for GHD children in terms of compliance and cost. However, the study failed to establish non-inferiority of biweekly PEG-rhGH *versus* weekly PEG-rhGH or rhGH with regard to HtSDS_CA_ and annualized HV. Nevertheless, the HV of group QOW reached 76.42%–90.34% that of the group QW and 76.08%–90.60% of the group QD, suggesting that with a less frequent dosing schedule and lowered medication cost, the once-biweekly dosing regimen still yields clinically meaningful improvement in HV. In the study, children receiving PEG-rhGH every other week had the smallest increase in median IGF-1 SDS at week 26, which is consistent with changes from baseline in HtSDS_CA_ at week 26 in comparison with weekly PEG-rhGH or rhGH. In addition, the smaller increase in IGF-1 SDS indicated that serum IGF-1 dynamics in children with GHD differs from that in healthy adults (as suggested in previous phase I trial), which is probably due to higher physiological GH and greater metabolic activities in children (9). It remains to be investigated whether other dosing intervals (such as 10 days) could offer comparative efficacy to once-weekly PEG-rhGH in children with GHD.

Safety remains a particularly important issue in view of potentially lifelong rhGH replacement therapy. The study showed a numerically lower incidence of AEs for biweekly PEG-rhGH (27.96%) than weekly PEG-rhGH (39.68%) and rhGH (33.3%). There were no new treatment-emergent AEs, and no children withdrew due to AEs. These findings are consistent with previous studies (10, 11). Furthermore, the study found no significant increase in AEs related to fluid retention, and glucose metabolism parameters were within the normal range in all three groups (Elevated serum IGF-I levels have been associated with increased incidence of AEs related to fluid retention and deterioration of glucose metabolism). However, IGF-1 SDS remained within the normal range through the course of treatment, and no worrisome increase of IGF-1 SDS was observed. Furthermore, one of the concerns about prolonged exposure to rhGH is possible worsening of insulin sensitivity and glucose metabolism. However, we observed no abnormalities in glucose metabolism parameters such as HbA1c and FPG with biweekly PEG-rhGH over the course of treatment, and no new cases of diabetes were reported, which are consistent with previous studies of long-acting GH formulations (12, 13).

One strength of the study is that we studied the effect of rhGH on IGF-2 and IGFBP-2. Although recent follow-up of children treated with rhGH did not identify the association between rhGH administration and long-term cancer risk in GHD patients, the nature of growth hormone as a cancer promoter is often criticized during clinical use (14). Among many biomarkers of cancer, IGF-2 and IGFBP-2 expression is upregulated in many types of cancer and is associated with an increased risk of developing cancers (15–17) therefore frequently considered as neoplastic marker (18–20). No studies have been conducted to measure changes in IGF-2 and IGFBP-2 levels in prepubertal GHD children receiving long-acting rhGH. In the current study, we demonstrated that PEG-rhGH therapy did not alter serum IGF-2 levels, while serum IGFBP-2 levels declined after 26 weeks of treatment. The findings from our study of 585 children over 26 weeks of GH therapy provide support to the safety of PEG-rhGH in GHD children.

Our study has limitations. First, besides height, HV, and bone age, we used the stimulated GH cutoff value at 10 ng/ml, which is adopted in the Chinese guideline (21) in current study for GHD diagnosis. The cutoff value increased the sensitivity; however, it reduced the specificity to include true GHD when compared with the cutoffs of 5 or 7 ng/ml (22), especially when the patient was not triaged by a low IGF-1 (<−1 SDS) (23). In our cohort, not all the patients demonstrated low IGF-1 levels, and this may be caused by the high peak GH cutoff value or inaccurate of GH provocative methods (24). Thus, the patients in our cohort could be heterogeneous, and other short stature, such as idiopathic short stature and mild skeletal dysplasia, could also be included; this might undermine the efficacy of GH therapy. However, the relatively big sample as a nationwide phase IV study provides the first meaningful efficacy and safety results in a near-real-world situation. Second, the current phase IV trial was an open-label study, and blinding was not possible given the difference in dosing interval. Third, the per-protocol analysis could conceal the actual efficacy of PEG-rhGH with longer interval and ideally improved compliance. Nevertheless, safety data for 26 weeks were reported, and long-term safety of PEG-rhGH has not been addressed. In addition, the study did not examine adherence rate of the subjects, which remains an important issue in improving long-term treatment outcomes. Lastly, due the large number of collected blood samples, there existed the possibility for occasional suboptimized sample disposal that could undermine the accuracy of the test results.

In conclusion, PEG-rhGH every other week improves HtSDS_CA_ and enhances annualized HV in Chinese children with GHD, with a safety profile comparable with that of weekly PEG-rhGH and daily rhGH. The tendency of a steady decrease in oncogenic IGF-2 and IGFBP-2 highlights the safety of long-acting rhGH exposure current study protocol. Though at the level of 80% growth promotion efficacy the study failed to establish non-inferiority of PEG-rhGH every other week *versus* the weekly PEG-rhGH or daily rhGH, PEG-rhGH every other week leads to clinically meaningful improvement in linear growth of GHD children, and further optimization of doses and dosing intervals of PEG-rhGH should be explored so that better treatment option can be provided to GHD children.

## Data Availability Statement

The raw data supporting the conclusions of this article will be made available by the authors, without undue reservation.

## Ethics Statement

The studies involving human participants were reviewed and approved by Children’s Hospital of Fudan University General Hospital of Ningxia Medical University Maternal and Child Health-Care Hospital in Guiyang Maternal and Child Health Care Hospital of Hainan Province Children’s Hospital affiliated to Zhengzhou University, Henan Children’s Hospital Lu’an People’s Hospital Peking Union Medical College Hospital, State Key Laboratory of Complex Severe and Rare Diseases, NMPA Key Laboratory for Clinical Research and Evaluation of Drug, Beijing Key Laboratory of Clinical PK & PD Investigation for Innovative Drugs, Chinese Academy of Medical Sciences & Peking Union Medical College The Second Affiliated Hospital of Nanchang University The Affiliated Suzhou Hospital of Nanjing Medical University Jinhua Hospital, Zhejiang University School of Medicine Jiaxing First Hospital Tai’an Maternal and Child Health Care Hospital Jiangsu Province Hospital of Chinese Medicine Second Hospital of Lanzhou University The First People’s Hospital of Changzhou The First Affiliated Hospital of Henan University of Science and Technology Inner Mongolia People’s Hospital Jishuitan Hospital Anhui Province Maternity and Child Health Hospital, and Anhui Medical University Maternal and Child Health Clinic College Hebei General Hospital Cixi People’s Hospital Affiliated Hospital of Nantong University Chengdu Children’s Specialized Hospital. Written informed consent to participate in this study was provided by the participants’ legal guardian/next of kin.

## Author Contributions

FL and RC conceived the study design, managed the study, conducted the data analysis, and wrote the manuscript. CS helped with the data analysis and editing of the manuscript. BL and YL contributed to the design of the study protocol, training of trial investigators. LX, BL, YL, YZ, HW, XH, PH, QZ, YLL, KY, KW, ZG, ZL, JY, HXZ, HZ, ZJ, YJL, NW, CY, JYY, LY, FH, and QY are site investigators and conducted the study in each participating center. All authors contributed to the article and approved the submitted version.

## Funding

The study was supported by GeneScience Pharmaceuticals Co., Ltd (Changchun, China). The sponsor was not involved in study implementation; in the collection, analysis, and interpretation of data; in the writing of the report; or in the decision to submit the article for publication.

## Conflict of Interest

CS, RC, and FL received lecture fees from GeneScience Pharmaceuticals.

The remaining authors declare that the research was conducted in the absence of any commercial or financial relationships that could be construed as a potential conflict of interest.

## Publisher’s Note

All claims expressed in this article are solely those of the authors and do not necessarily represent those of their affiliated organizations, or those of the publisher, the editors and the reviewers. Any product that may be evaluated in this article, or claim that may be made by its manufacturer, is not guaranteed or endorsed by the publisher.

## Appendix I. List of participating institutions

1 Children’s Hospital of Fudan University, 399 Wan Yuan Road, Shanghai 201102, China.

2 General Hospital of Ningxia Medical University, Yinchuan 750004, Ningxia Hui Autonomous Region, China.

3 Maternal and Child Health-Care Hospital in Guiyang, Guiyang 550003, Guizhou Province, China.

4 Maternal and Child Health Care Hospital of Hainan Province, Haikou 570206, Hainan Province, China.

5 Children’s Hospital affiliated to Zhengzhou University, Henan Children’s Hospital, Zhengzhou 450018, Henan Province, China.

6 Lu’an People’s Hospital, Lu’an 237000, Anhui Province, China.

7 Peking Union Medical College Hospital, State Key Laboratory of Complex Severe and Rare Diseases, NMPA Key Laboratory for Clinical Research and Evaluation of Drug, Beijing Key Laboratory of Clinical PK & PD Investigation for Innovative Drugs, Chinese Academy of Medical Sciences & Peking Union Medical College, Beijing 100032, China.

8 The Second Affiliated Hospital of Nanchang University, Nanchang 330006, Jiangxi Province, China.

9 The Affiliated Suzhou Hospital of Nanjing Medical University, Suzhou 215002, Jiangsu Province, China.

10 Jinhua Hospital, Zhejiang University School of Medicine, Jinhua 321000, Zhejiang Province, China.

11 Jiaxing First Hospital, Jiaxing 314000, Zhejiang Province, China.

12 Tai’an Maternal and Child Health Care Hospital, Tai’an 271000, Shandong Province, China.

13 Jiangsu Province Hospital of Chinese Medicine, Nanjing 210029, Jiangsu Province, China.

14 Second Hospital of Lanzhou University, Lanzhou 730030, Gansu Province, China.

15 The First People’s Hospital of Changzhou, Changzhou 213000, Jiangsu Province, China.

16 The First Affiliated Hospital of Henan University of Science and Technology, Luoyang 471003, Henan Province, China.

17 Inner Mongolia People’s Hospital, Hohhot 010017, Inner Mongolia Autonomous Region, China.

18 Jishuitan Hospital, Beijing 100035, China.

19 Anhui Province Maternity and Child Health Hospital, Anhui Medical University Maternal and Child Health Clinic College, Hefei 230001, Anhui Province, China.

20 Hebei General Hospital, Shijiazhuang 050051, Hebei Province, China.

21 Cixi People’s Hospital, Cixi 315300, Zhejiang Province, China.

22 Affiliated Hospital of Nantong University, Nantong 226000, Jiangsu Province, China.

23 Chengdu Children’s Specialized Hospital, Chengdu 610015, Sichuan Province, China.
