# Corrigendum: Reduced Effectiveness and Comparable Safety in Biweekly *vs.* Weekly PEGylated Recombinant Human Growth Hormone for Children With Growth Hormone Deficiency: A Phase IV Non-Inferiority Threshold Targeted Trial

**DOI:** 10.3389/fendo.2021.830469

**Published:** 2021-12-28

**Authors:** Chengjun Sun, Biao Lu, Yu Liu, Yaqin Zhang, Haiyan Wei, Xu Hu, Pei Hu, Qian Zhao, Yanling Liu, Kan Ye, Kan Wang, Zaiyan Gu, Zheng Liu, Jin Ye, Hongxiao Zhang, Hong Zhu, Zhihong Jiang, Yanjie Liu, Naijun Wan, Chengming Yan, Jianying Yin, Lirong Ying, Feng Huang, Qingjin Yin, Li Xi, Feihong Luo, Ruoqian Cheng

**Affiliations:** ^1^ Department of Pediatric Endocrinology and Inherited Metabolic Diseases, Children’s Hospital of Fudan University, Shanghai, China; ^2^ Department of Pediatrics, General Hospital of Ningxia Medical University, Yinchuan, China; ^3^ Department of Endocrine and Genetic Metabolism, Maternal and Child Health-Care Hospital in Guiyang, Guiyang, China; ^4^ Department of Child Health, Maternal and Child Health Care Hospital of Hainan Province, Haikou, China; ^5^ Department of Pediatric Endocrinology and Inherited Metabolic Diseases, Henan Provincial Hospital, Affiliated to Zhengzhou University, Zhengzhou, China; ^6^ Department of Pediatrics, Lu’an People’s Hospital, Lu’an, China; ^7^ Clinical Pharmacology Research Center, Peking Union Medical College Hospital, State Key Laboratory of Complex Severe and Rare Diseases, National Medical Products Administration (NMPA) Key Laboratory for Clinical Research and Evaluation of Drug, Beijing Key Laboratory of Clinical Pharmacokinetics and Pharmacodynamics (PK & PD) Investigation for Innovative Drugs, Chinese Academy of Medical Sciences & Peking Union Medical College, Beijing, China; ^8^ Department of Pediatrics, The Second Affiliated Hospital of Nanchang University, Nanchang, China; ^9^ Department of Child Health, The Affiliated Suzhou Hospital of Nanjing Medical University, Suzhou, China; ^10^ Department of Pediatrics, Jinhua Hospital, Zhejiang University School of Medicine, Jinhua, China; ^11^ Department of Pediatrics, Jiaxing First Hospital, Jiaxing, China; ^12^ Department of Pediatrics, Tai’an Maternal and Child Health Care Hospital, Tai’an, China; ^13^ Department of Pediatrics, Jiangsu Province Hospital of Chinese Medicine, Nanjing, China; ^14^ Department of Pediatrics, Second Hospital of Lanzhou University, Lanzhou, China; ^15^ Department of Pediatrics, The First People’s Hospital of Changzhou, Changzhou, China; ^16^ Department of Pediatrics, The First Affiliated Hospital of Henan University of Science and Technology, Luoyang, China; ^17^ Department of Pediatrics, Inner Mongolia People’s Hospital, Hohhot, China; ^18^ Department of Pediatrics, Jishuitan Hospital, Beijing, China; ^19^ Department of Pediatrics, Anhui Province Maternity and Child Health Hospital, Anhui Medical University Maternal and Child Health Clinic College, Hefei, China; ^20^ Department of Pediatrics, Hebei General Hospital, Shijiazhuang, China; ^21^ Department of Pediatrics, Cixi People’s Hospital, Cixi, China; ^22^ Department of Pediatrics, Affiliated Hospital of Nantong University, Nantong, China; ^23^ Department of Internal Medicine, Chengdu Children’s Specialized Hospital, Chengdu, China

**Keywords:** growth hormone deficiency, PEGylated recombinant human growth hormone, PEG-rhGH, IGF-2, children

The second and third authors, Biao Lu and Yu Liu, were not listed as co-first-authors in the article as published originally.

The Author Contributions statement, published originally read “FL and RC conceived the study design, managed the study, conducted the data analysis, and wrote the manuscript. CS helped with the data analysis and editing of the manuscript. LX, BL, YL, YZ, HW, XH, PH, QZ, YLL, KY, KW, ZG, ZL, JY, HXZ, HZ, ZJ, YJL, NW, CY, JYY, LY, FH, and QY are site investigators and conducted the study in each participating center. All authors contributed to the article and approved the submitted version”. The corrected Author Contributions statement is as follows: “FL and RC conceived the study design, managed the study, conducted the data analysis, and wrote the manuscript. CS helped with the data analysis and editing of the manuscript. BL and YL contributed to the design of the study protocol, training of trial investigators. LX, BL, YL, YZ, HW, XH, PH, QZ, YLL, KY, KW, ZG, ZL, JY, HXZ, HZ, ZJ, YJL, NW, CY, JYY, LY, FH, and QY are site investigators and conducted the study in each participating center. All authors contributed to the article and approved the submitted version.”

The authors apologize for this error and state that this does not change the scientific conclusions of the article in any way. The original article has been updated.

## Publisher’s Note

All claims expressed in this article are solely those of the authors and do not necessarily represent those of their affiliated organizations, or those of the publisher, the editors and the reviewers. Any product that may be evaluated in this article, or claim that may be made by its manufacturer, is not guaranteed or endorsed by the publisher.

