# Supplementary material for: Reduced Effectiveness and Comparable Safety in Biweekly vs. Weekly PEGylated Recombinant Human Growth Hormone for Children With Growth Hormone Deficiency: A Phase IV Non-Inferiority Threshold Targeted Trial
Source: Front Endocrinol (Lausanne). 2021 Nov 25;12:779365. doi: 10.3389/fendo.2021.779365 (PMC8655095; doi:10.3389/fendo.2021.779365)
Supplement: Supplementary file 1 [file DataSheet_1.docx]

**Supplementary Table 1** ANCOVA of changes from baseline in Ht SDS_CA_, Ht SDS_BA_, BMI SDS, IGF-1 SDS, and IGF-1/IGFBP-3 molar ratio at week 26 in the FAS

| Factors | Ht SDS_CA_ | Ht SDS_BA_ | BMI SDS | IGF-1 SDS | IGF-1/IGFBP-3 molar ratio |
| --- | --- | --- | --- | --- | --- |
| Least square mean (95%CI) |  |  |  |  |  |
| Group QW | 0.560(0.516,0.603) | 0.310(0.073,0.546) | 0.221(0.130,0.313) | 1.610(1.259,1.961) | 0.050(0.036,0.065) |
| Group QOW | 0.382(0.338,0.425) | 0.381(0.148,0.614) | 0.095(0.003,0.187) | 0.858(0.516,1.200) | 0.032(0.017,0.046) |
| Group QD | 0.544(0.499,0.588) | 0.402(0.165,0.639) | -0.091(-0.184,0.002) | 0.959(0.596,1.321) | 0.033(0.018,0.048) |
| Difference (QW-QOW) | 0.178(0.106,0.251) | -0.071(-0.370,0.227) | 0.126(0.001,0.251) | 0.752(0.297,1.207) | 0.019(-0.000,0.038) |
| Difference (QW-QD) | 0.016(-0.057,0.089) | -0.092(-0.397,0.212) | 0.312(0.186,0.439) | 0.652(0.187,1.117) | 0.017(-0.002,0.036) |
| Difference (QOW-QD) | -0.162(-0.236,-0.089) | -0.021(-0.323,0.281) | 0.187(0.060,0.313) | -0.101(-0.560,0.359) | -0.002(-0.021,0.017) |
| Factor-group |  |  |  |  |  |
| Effect | 1.604 | 0.299 | 4.019 | 14.805 | 0.008 |
| F | 21.233 | 0.198 | 11.981 | 6.159 | 2.244 |
| P | <0.001 | 0.820 | 0.000 | 0.002 | 0.109 |
| Factor-baseline |  |  |  |  |  |
| Effect | 2.515 | 84.529 | 71.069 | 125.745 | 2.379 |
| F | 33.298 | 55.946 | 211.860 | 52.313 | 656.529 |
| P | <0.001 | <0.001 | 0.000 | <0.001 | <0.001 |
| Factor-center |  |  |  |  |  |
| Effect | 0.115 | 3.215 | 0.500 | 3.580 | 0.006 |
| F | 1.521 | 2.128 | 1.492 | 1.490 | 1.526 |
| P | 0.066 | 0.004 | 0.075 | 0.085 | 0.083 |

**Supplementary Table 2** Stepwise multivariate logistic regression analysis of determinants of Ht SDS_CA_ change at week 26

| Variables | Non-standardized coefficients | | Standardized coefficients | t | *p* |
| --- | --- | --- | --- | --- | --- |
|  | B | Standard error |  |  |  |
| Constant | 0.917 | 0.039 | 0 | 23.67 | <0.001 |
| Group |  |  |  |  |  |
| QW *vs.* QOW | 0.135 | 0.021 | 0.341 | 6.40 | <0.001 |
| QD *vs.* QOW | 0.117 | 0.022 | 0.292 | 5.42 | <0.001 |
| Age | -0.022 | 0.003 | -0.342 | -6.54 | <0.001 |
| Baseline IGF-1 | -0.0003 | 0.0001 | -0.168 | -3.49 | 0.001 |
| GH peak | -0.013 | 0.004 | -0.181 | -3.80 | <0.001 |

**Supplementary Table 3** Adverse events (AEs) with an incidence of at least 2% in the safety set

| AEs | QW  (N=189) | | QOW  (N=186) | | QD  (N=177) | | All (N=552) | |
| --- | --- | --- | --- | --- | --- | --- | --- | --- |
|  | n | % | n | % | n | % | n | % |
| Upper respiratory tract infection | 51 | 26.98% | 57 | 30.65% | 55 | 31.07% | 163 | 29.53% |
| Fever | 12 | 6.35% | 21 | 11.29% | 36 | 20.34% | 69 | 12.50% |
| Cough | 18 | 9.52% | 9 | 4.84% | 3 | 1.69% | 30 | 5.43% |
| Bronchial infection | 3 | 1.59% | 10 | 5.38% | 4 | 2.26% | 17 | 3.08% |
| Abdominal pain | 4 | 2.12% | 4 | 2.15% | 2 | 1.13% | 10 | 1.81% |
| Joint pain | 3 | 1.59% | 4 | 2.15% | 1 | 0.56% | 8 | 1.45% |
| Injection site reaction | 4 | 2.12% | 4 | 2.15% | 0 | 0.00% | 8 | 1.45% |
| Pneumonia | 4 | 2.12% | 2 | 1.08% | 0 | 0.00% | 6 | 1.09% |
| Muscle pain | 5 | 2.65% | 0 | 0.00% | 0 | 0.00% | 5 | 0.91% |
